# Optical character recognition system for Baybayin scripts using support vector machine

**DOI:** 10.7717/peerj-cs.360

**Published:** 2021-02-15

**Authors:** Rodney Pino, Renier Mendoza, Rachelle Sambayan

**Affiliations:** Institute of Mathematics, University of the Philippines Diliman, Quezon City, Metro Manila, Philippines

**Keywords:** Baybayin, Latin script identification, Baybayin script identification, Support vector machine, Optical character recognition

## Abstract

In 2018, the Philippine Congress signed House Bill 1022 declaring the Baybayin script as the Philippines’ national writing system. In this regard, it is highly probable that the Baybayin and Latin scripts would appear in a single document. In this work, we propose a system that discriminates the characters of both scripts. The proposed system considers the normalization of an individual character to identify if it belongs to Baybayin or Latin script and further classify them as to what unit they represent. This gives us four classification problems, namely: (1) Baybayin and Latin script recognition, (2) Baybayin character classification, (3) Latin character classification, and (4) Baybayin diacritical marks classification. To the best of our knowledge, this is the first study that makes use of Support Vector Machine (SVM) for Baybayin script recognition. This work also provides a new dataset for Baybayin, its diacritics, and Latin characters. Classification problems (1) and (4) use binary SVM while (2) and (3) apply the multiclass SVM classification. On average, our numerical experiments yield satisfactory results: (1) has 98.5% accuracy, 98.5% precision, 98.49% recall, and 98.5% F1 Score; (2) has 96.51% accuracy, 95.62% precision, 95.61% recall, and 95.62% F1 Score; (3) has 95.8% accuracy, 95.85% precision, 95.8% recall, and 95.83% F1 Score; and (4) has 100% accuracy, 100% precision, 100% recall, and 100% F1 Score.

## Introduction

Baybayin is one of the pre-Hispanic writing systems used in the Philippines (Cabuay, 2009). In 2018, the Philippine government is showing efforts to preserve and reintroduce this heritage, mandating all local government units to inscribe Baybayin script with its translation in their communication systems (e.g., signage), through House Bill 1022. Furthermore, local manufacturers are required to use Baybayin on labels, and the Education Department is tasked to promote the said writing system (Lim & Manipon, 2019).

**Figure 1:**
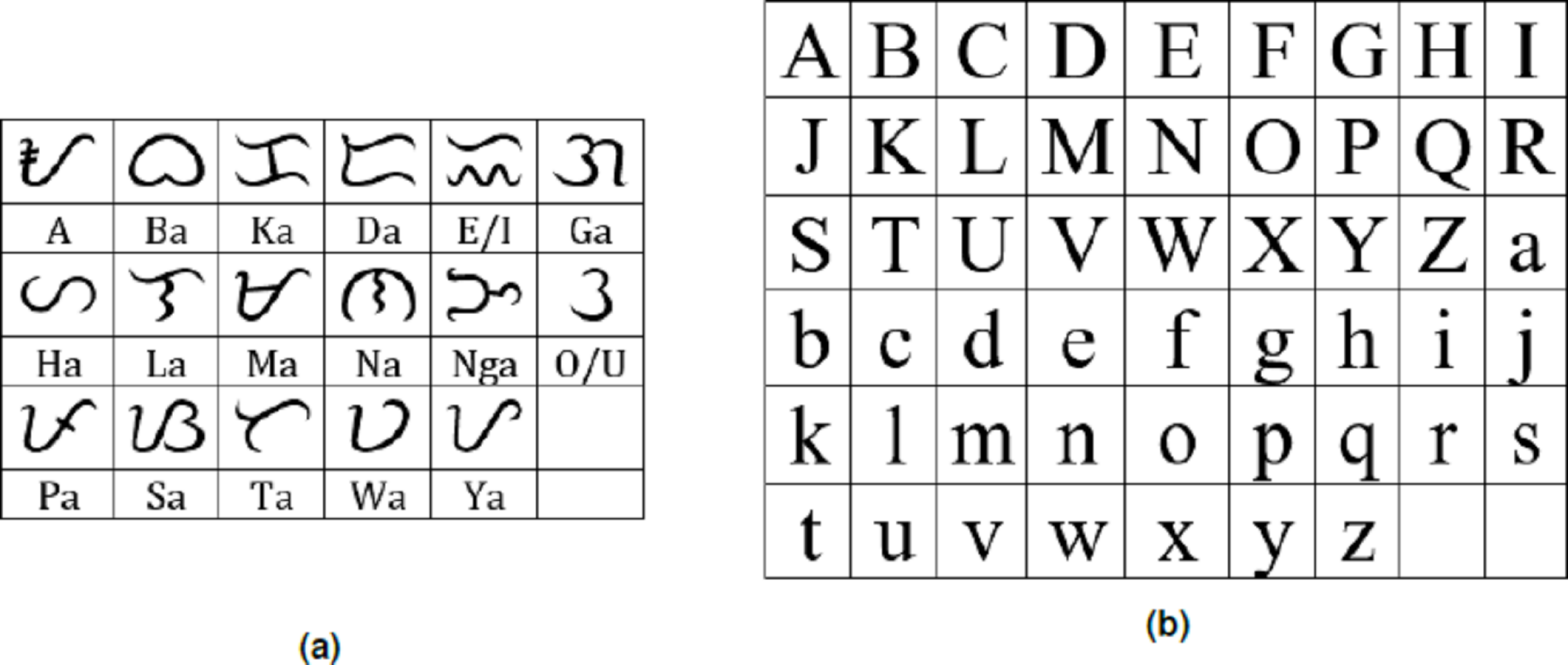
(A) Baybay in characters (without diacritics) with equivalent Latin syllable; (B) Latin alphabet in upper- and lowercase.

**Figure 2:**
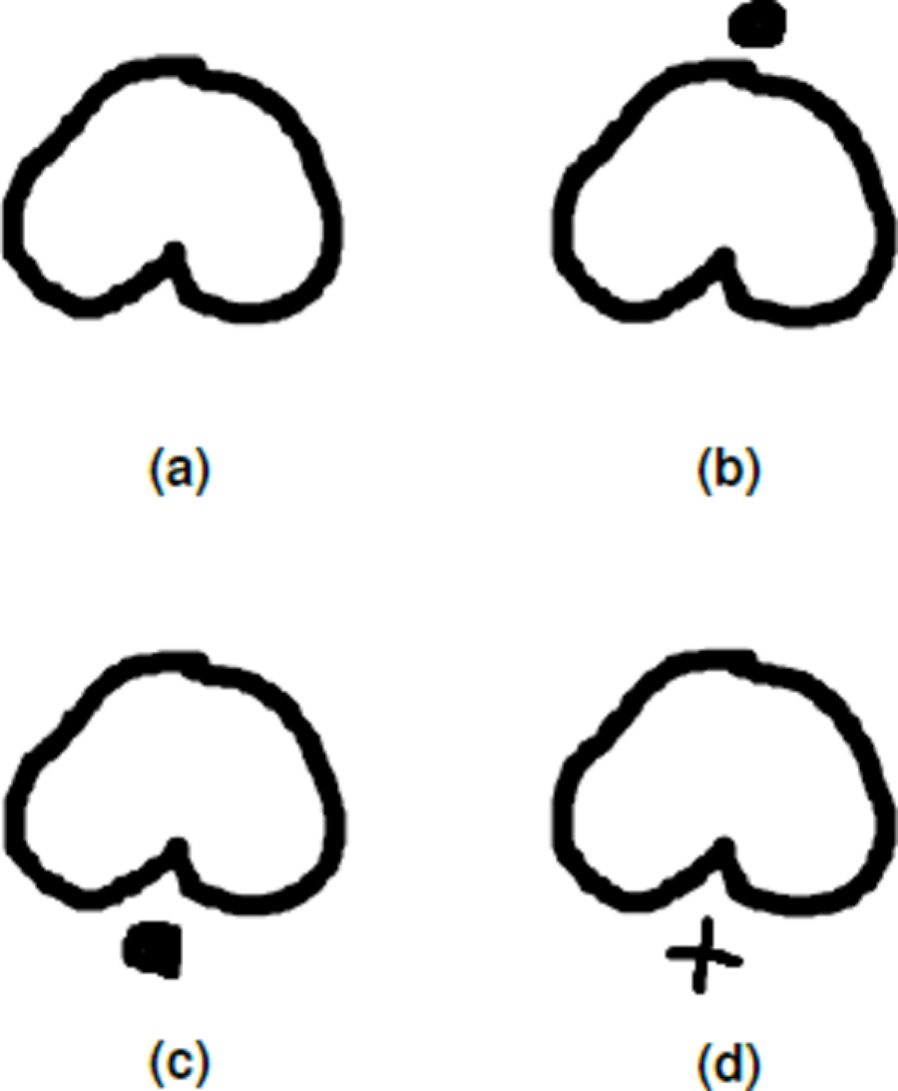
Incorporating diacritics in a Baybayin (consonant) character. The dot diacritic changes the ‘Ba’ character in A into ‘Be’ or ‘Bi’ character if written above the character (B), and into ‘Bo’ or ‘Bu’ if written below the character (C). A cross diacritic written below the ‘Ba’ character cancels the vowel ‘a’ (D).

Baybayin is an abugida, or alphasyllabary, primarily used by the Tagalogs in northern Philippines during the pre-colonial period. Baybayin consists of 17 unique characters: 14 (syllabic) consonants and three vowels (see Fig. 1A). In Baybayin, consonants are pronounced with an inherent vowel sound ‘ ∖a∖’, and diacritics are used to express the other vowels. For example, a diacritic written above a consonant character may represent an accompaniment vowel ‘ ∖e∖’ or ‘ ∖i∖’, while a diacritic written below may represent an ‘ ∖o∖’ or ‘ ∖u∖’ sound. Diacritics can also be used to silence the vowel sounds. Figure 2 shows an example of the phonetic distinction in a Baybayin consonant character using diacritical marks. While there are no standard accent symbols for Baybayin script, the most commonly used are bar, dot, cross, or x. A bar or a dot represents the vowels E/I or O/U based on their placement, while the cross or x symbol located below the character cancels the vowel “a” (Cabuay, 2009).

Optical Character Recognition (OCR) is a process of reading and recognizing handwritten or printed characters. It belongs to the family of machine recognition techniques where the system performs an automatic identification of scripts and characters (Chaudhuri et al., 2017).

Prior to choosing an appropriate character recognition algorithm, it is important to determine first the script used in writing a document. Most research studies in script identification have considered printed and handwritten scripts, and are based on several levels: page, line, word, and character level (Ghosh, Dube & Shivaprasad, 2010). With English as a global language and Latin as its standard script, there have been a lot of script identification techniques that discriminate the Latin script from other writing systems. Jaeger, Ma & Doermann (2005) have identified the Latin script from Arabic, Chinese, Hindi, and Korean scripts using modified document spectrum algorithm and four classifier systems at word level. At text block level script identification, Zhou, Lu & Tan (2006) have distinguished Bangla against Latin script using connected component analysis. Several studies by Chanda, Pal & Kimura (2007) about script recognition include: identifying Japanese from Latin script using a single text line scheme based on tree classifier, recognizing Thai from Latin script using a single text line scheme based on Support Vector Machine (SVM) (Chanda, Terrades & Pal, 2007), and recognizing Latin from all Han-based scripts (Chinese, Japanese, and Korean) via a multi-script OCR system that uses chain-code histogram-based directional features and SVM at character and block level (Chanda et al., 2010). At character level, Zhu et al. (2009) have reported recognition of Chinese script from Latin script using feature selection and cascade classifier, while Rani, Dhir & Lehal (2013) have proposed a technique using Gabor filter and gradient-based features using SVM classifier to recognize Gurumukhi and Latin scripts. At word level, on the other hand, Hangarge, Santosh & Pardeshi (2013) have reported script recognition based on directional discrete cosine transforms (D-DCT) to six Indic scripts, namely, Devanagari, Kannada, Telugu, Tamil, Malayalam, and Latin. Rajput & Ummapure (2017) have employed Scale Invariant Feature Transportation (SIFT) approach and *K* −Nearest Neighbor (KNN) classifier to identify Latin, Kannada, and Devanagari scripts. Multi-script identification at page level based on features extracted via Structural and Visual Appearance (SVA) and Directional Stroke Identification (DSI) has been proposed by Obaidullah et al. (2017). Recognition of eleven Indian scripts together with the Latin script has been carried out using Multilayer Perceptron (MLP) and Simple Logistic (SL) classifiers. Script identification for Chinese, Kannada, Greek, Latin, and other scripts in natural scene text images and video scripts has been done by extracting global and local features, with the use of attention-based Convolutional Neural Network (CNN) and Long Short-Term Memory (LSTM) Network (Bhunia, Konwer & Bhunia, 2019).

Among the many varieties of the OCR algorithm, the SVM classifier is one of the most popular because of its high response speed, robustness, and good accuracy (Thomé, 2012). The SVM algorithm was first introduced by Vladimir Vapnik and Alexey Chervonenkis in 1963. It belongs to the family of supervised classification techniques based on statistical learning theory. SVM algorithm has been very well-developed for the past decade where it seeks the optimal hyperplane that maximizes the margins between the borders of two classes (Thomé, 2012). For an extensive discussion on how SVM works, we refer the readers to Cristianini & Shawe-Taylor (2000); Shawe-Taylor & Cristianini (2004); Bishop (2006), and Schölkopf & Smola (2012). Several applications of SVM include face and object detection and recognition, image information retrieval, time series prediction (Sapankevych & Sankar, 2009), speech recognition (Ganapathiraju, Hamaker & Picone, 2004), data mining (Nayak, Naik & Behera, 2015), bioinformatics (Yang, 2004; Rivero, Lemence & Kato, 2017; Rivero & Kato, 2018; Do & Le, 2019), and genomics (Le et al., 2019; Le, 2019). Among these many applications, SVM is reported to greatly outperform most of the other learning algorithms for handwritten character and digit recognition problems (Byun & Lee, 2003).

Many script character recognition systems have been reported with SVM as classifiers. In their experiments, Phangtriastu, Harefa & Tanoto (2017) have shown that SVM can achieve an accuracy of 94.43%, and is better when compared to Artificial Neural Network. Tautu & Leon (2012) have studied how SVM can be used to classify handwritten Latin letters, and obtained over 90% precision when tests were implemented on small or capital Latin letters. Sok & Taing (2014) have proposed SVM for printed Khmer Characters and obtained 98.62% recognition accuracy. Using SVM models, Shanthi & Duraiswamy (2009) have reported recognition for Tamil characters and obtained 82.04% accuracy. With 96.79% recognition rate, identification of Arabic handwritten characters have been presented by Althobaiti & Lu (2018) with the use of SVM classifiers and Normalized Central Moments (NCM) and Local Binary Patterns (LBP) for feature extraction. Aggarwal & Singh (2015) have reported gradient and curvature approach in feature extraction and have made use of SVM models in recognizing Gurmukhi characters where they obtained an accuracy of 98.56%. Using Zernike invariants and SVM classifiers, Kaushal, Khan & Varma (2014) have proposed a technique in identifying Urdu characters and obtained a recognition accuracy of 96.29%. Dong, Krzyak & Suen (2005) have used directional histograms and SVM models to recognize Chinese characters. Their work yields a 99% recognition rate. An accuracy of 87.32% have been reported by Kilic et al. (2008) using SVM model for Ottoman character recognition. Recognition of Malayalam characters using wavelet transforms and SVM classifiers have been introduced by John, Pramod & Balakrishnan (2012), where their empirical results obtained a 90.25% accuracy. Gaur & Yadav (2015) have performed *K*-means clustering and used SVM classifiers to recognize Hindi characters and obtained a result of 95.86% accuracy. Utilizing SVM and combining Zoning and Gabor filter in feature extraction yields a result of 92.99% recognition rate in classifying Bangla characters (Pervin, Afroge & Huq, 2017).

Baybayin OCR is still in its infancy in literature. There are only few mathematical studies on Baybayin script. Recario et al. (2011) have presented an automated reader for Baybayin scripts where the system outputs the equivalent syllables of the Baybayin character. They used the Line Angle Categorization and Freeman Chain Coding for classification, and obtained results of 51.96% and 66.47% recognition accuracy, respectively. Nogra, Romana & Maravillas (2019) and Nogra, Romana & Balakrishnan (2020) have proposed an LSTM neural network and CNN models, with 92.90% and 94.00% recognition rates, respectively, that translates Baybayin handwritten characters to their corresponding syllabic unit in Latin alphabet. While their works were done at character level, they have greatly contributed in introducing Baybayin characters to computer vision. Recio & Mendoza (2019) have implemented an edge detection approach on recognizing old images containing Baybayin texts. The method has been generally shown to be effective in detecting the texts’ edges and reducing the noise level of an old image.

In line with the restoration of the script, we propose an OCR system that distinguishes Baybayin from the Latin script at character level in either handwritten or computer-generated form. The 17 main Baybayin characters and 52 (26 each for upper and lower cases) Latin characters are shown in Fig. 1. In this paper, we introduce a technique in classifying the characters of both scripts with the aid of SVM.

The remainder of this paper is organized as follows: in ‘A Review of Support Vector Machine’, a brief review of how support vector machine works is presented. ‘Collecting Baybayin Dataset’ discusses how Baybayin, its accents, and Latin characters were gathered. The proposed OCR algorithm for Baybayin and Latin script and character recognition are presented in ‘Proposed OCR System’. In ‘Experimental Setup, Results and Discussions’, the results and discussion of our proposed OCR algorithm are shown. Lastly, conclusions and future works are presented in ‘Conclusions and Future Works’.

## A Review of Support Vector Machine

Our proposed OCR system consists of four classification problems wherein SVM is used. SVM considers a training set of points ${\vec{x}}_{i}\in {\mathbb{R}}^{n},$i=1 , …, *N*, where *n* is the number of features in a particular training sample and *N* is the number of training points. In a two-class or binary classification problem, each of these points are labeled by an indicator variable *y*_*i*_ ∈ { − 1, 1}, depending on the class in which the data point belongs. The points are separated by classes or categories by a hyperplane called a *linear classifier*. The linear classifier can be written with a set of points $\vec{x}$ satisfying

(1) \begin{eqnarray*}\vec{w}\cdot \vec{x}+b=0,\end{eqnarray*}

where $\vec{w}$ and *b* are the weight vector and bias term, respectively. With vector $\vec{w}$ normal to the hyperplane (Eq. (1)), the distance of the hyperplane from the origin is ${|}b{|}/\parallel \vec{w}\parallel $.

**Figure 3:**
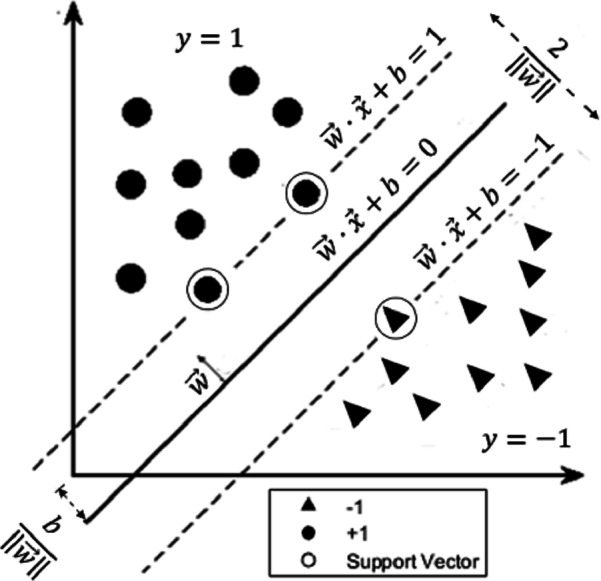
The maximum-margin hyperplane. The figure shows an example of a two-class data separated by a linear classifier. The parallel dashed lines represent the hyperplanes $\vec{w}\cdot \vec{x}+b=1$ and $\vec{w}\cdot \vec{x}+b=-1$ where the distance between them is $ \frac{2}{\parallel \vec{w}\parallel } $. The circular-shaped data belong to the positive group (labeled with *y* = 1) while the triangular-shaped data belong to the negative group (labeled with *y* =  − 1). The points on the dashed lines are called support vectors. SVM finds the optimal hyperplane (solid line) whose distance from the origin is $ \frac{b}{\parallel \vec{w}\parallel } $ and lies halfway between the two (dashed) hyperplanes.

In a linearly separable case (i.e., it is possible to draw a line that can separate the two classes), we can select two parallel hyperplanes (dashed lines in Fig. 3) that separate the two classes with maximum distance from each other, in a way that there are no data points in between. The linear classifier (Eq. (1)) lies halfway between these hyperplanes, and the region bounded by one of these hyperplanes and the linear classifier is called a *margin*. SVM finds the values for the weight and bias that gives the maximum-margin hyperplane that separates the two classes, so that any point on or above the hyperplane

\begin{eqnarray*}\vec{w}\cdot \vec{x}+b=1 \end{eqnarray*}

is labeled 1 while points on or below

\begin{eqnarray*}\vec{w}\cdot \vec{x}+b=-1 \end{eqnarray*}

is labeled −1. Hence, for any training data point ${\vec{x}}_{i}$ and its corresponding label *y*_*i*_, we have

\begin{eqnarray*}{y}_{i}(\vec{w}\cdot {\vec{x}}_{i}+b)\geq 1,i=1,\ldots ,N, \end{eqnarray*}

and the optimization problem is then given by

\begin{eqnarray*}\text{minimize}_{\vec{w},b}~~ \frac{1}{2} \parallel \vec{w}{\parallel }^{2} \end{eqnarray*}

\begin{eqnarray*}\text{subject to}~~{y}_{i} \left( \vec{w}\cdot {\vec{x}}_{i}+b \right) \geq 1  \text{for all}i=1,\ldots ,N. \end{eqnarray*}

The primal Lagrangian is constructed as

(2) \begin{eqnarray*}L \left( \vec{w},b,\vec{\alpha } \right) = \frac{1}{2} \parallel \vec{w}{\parallel }^{2}-\sum _{i=1}^{N}{\alpha }_{i} \left[ {y}_{i} \left( \vec{w}\cdot {\vec{x}}_{i}+b \right) -1 \right] ,\end{eqnarray*}

where *α*_1_, *α*_2_, …, *α*_*N*_ ≥ 0 are Lagrange multipliers. Differentiating (Eq. (3)) with respect to $\vec{w}$ and *b* and then equating to zero, yields

(3) \begin{eqnarray*}\vec{w}=\sum _{i=1}^{N}{y}_{i}{\alpha }_{i}{\vec{x}}_{i}\end{eqnarray*}

and

(4) \begin{eqnarray*}\sum _{i=1}^{N}{\alpha }_{i}{y}_{i}=0,\end{eqnarray*}

respectively. Substituting (Eqs. (4)) and ((5)) to ((3)), the primal Lagrangian (Eq. (3)) becomes

(5) \begin{eqnarray*}L \left( \vec{w},b,\vec{\alpha } \right) =\sum _{i=1}^{N}{\alpha }_{i}- \frac{1}{2} \sum _{i=1}^{N}\sum _{j=1}^{N}{y}_{i}{y}_{j}{\alpha }_{i}{\alpha }_{j}({\vec{x}}_{i}\cdot {\vec{x}}_{j}).\end{eqnarray*}

The Karush–Kuhn–Tucker (KKT) conditions assert that the optimal solutions ${\vec{\alpha }}^{\ast }$, ${\vec{w}}^{\ast }$, and *b*^∗^ must satisfy

(6) \begin{eqnarray*}{\alpha }_{i}^{\ast } \left[ {y}_{i} \left( {\vec{w}}^{\ast }\cdot {\vec{x}}_{i}+{b}^{\ast } \right) -1 \right] =0,\end{eqnarray*}

which implies that for all nonzero ${\vec{\alpha }}_{i}^{\ast }$,

\begin{eqnarray*}{y}_{i} \left( {\vec{w}}^{\ast }\cdot {\vec{x}}_{i}+{b}^{\ast } \right) =1, \end{eqnarray*}

where the ${\vec{x}}_{i}$’s are precisely the data points or vectors on the margin. These vectors are called *support vectors* as they are the ones which determine or “support” the margins. From (Eqs. (1)) and ((4)), the maximum-margin hyperplane is now given by

(7) \begin{eqnarray*}\sum _{i\in S}{y}_{i}{\alpha }_{i}^{\ast } \left( {\vec{x}}_{i}\cdot \vec{x} \right) +{b}^{\ast }=0,\end{eqnarray*}

where *S* is the set of indices of the support vectors. A new data point $\vec{x}\in {\mathbb{R}}^{n}$ can now be assigned a class through the decision function

(8) \begin{eqnarray*}f(\vec{x})=\text{sign} \left( \sum _{i\in S}{y}_{i}{\alpha }_{i}^{\ast } \left( {\vec{x}}_{i}\cdot \vec{x} \right) +{b}^{\ast } \right) ,\end{eqnarray*}

using only the support vectors.

**Figure 4:**
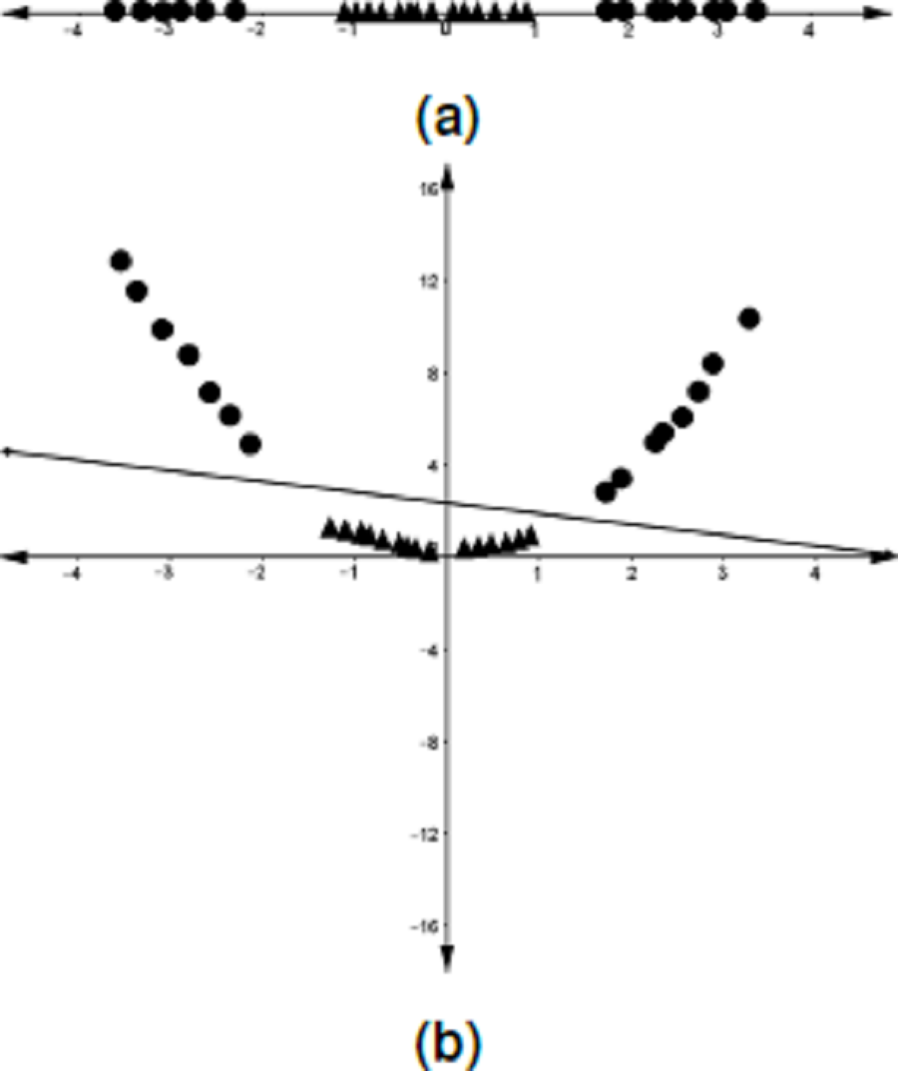
Mapping of the data points from the input space onto a feature space. (A) One-dimensional dataset that cannot be separated by a linear classifier. (B) The data points in A are mapped into a higher-dimensional space (feature space) where a linear classifier is found.

Although there are datasets that can be linearly classified, this is not usually the case, as can be seen in Fig. 4A. As a solution, Boser, Guyon & Vapnik (1992) proposed that the data in the input space be mapped into a higher-dimensional space, called a *feature space*, where a linear separator could be found (see Fig. 4B). The computation of the optimal hyperplane in this scenario is not done explicitly on the feature space but rather by the use of a *kernel trick*. This is briefly discussed below.

Suppose that the data points in the input space are mapped into a feature space by using some nonlinear function *ϕ*. Then (Eq. (6)) becomes

(9) \begin{eqnarray*}L=\sum _{i=1}^{N}{\alpha }_{i}- \frac{1}{2} \sum _{i=1}^{N}\sum _{j=1}^{N}{y}_{i}{y}_{j}{\alpha }_{i}{\alpha }_{j} \left( \phi ({\vec{x}}_{i})\cdot \phi ({\vec{x}}_{j}) \right) .\end{eqnarray*}

Similarly, (Eqs. (8)) and ((9)) become

(10) \begin{eqnarray*}\sum _{i\in S}{y}_{i}{\alpha }_{i}^{\ast } \left( \phi ({\vec{x}}_{i})\cdot \phi ({\vec{x}}_{j}) \right) +{b}^{\ast }=0,\end{eqnarray*}

and

(11) \begin{eqnarray*}f(\vec{x})=\text{sign} \left( \sum _{i\in S}{y}_{i}{\alpha }_{i}^{\ast } \left( \phi ({\vec{x}}_{i})\cdot \phi ({\vec{x}}_{j}) \right) +{b}^{\ast } \right) ,\end{eqnarray*}

respectively. Notice that in the above equations, the inner product happens only on the images of the inputs ${\vec{x}}_{i}$ and ${\vec{x}}_{j}$. Mercer’s theorem states that if,

(12) \begin{eqnarray*}\kappa \left( {\vec{x}}_{i},{\vec{x}}_{j} \right) =\phi ({\vec{x}}_{i})\cdot \phi ({\vec{x}}_{j})\end{eqnarray*}

is positive-definite, then the function *κ* in (Eq. (13)) is called a *kernel function*. Equation (13) also tells us that as long as the function *κ* is positive-definite, we are assured of some inner products of the images in the feature space. This follows that the coordinates of the images or mapped data points need not be explicitly computed, and the kernel trick allows us to write (Eqs. (10)), ((11)), and ((12)) as

(13) \begin{eqnarray*}L=\sum _{i=1}^{N}{\alpha }_{i}- \frac{1}{2} \sum _{i=1}^{N}\sum _{j=1}^{N}{y}_{i}{y}_{j}{\alpha }_{i}{\alpha }_{j}\kappa \left( {\vec{x}}_{i},{\vec{x}}_{j} \right) ,\end{eqnarray*}

(14) \begin{eqnarray*}\sum _{i\in S}{y}_{i}{\alpha }_{i}^{\ast }\kappa \left( {\vec{x}}_{i},{\vec{x}}_{j} \right) +{b}^{\ast }=0, \text{and}\end{eqnarray*}

(15) \begin{eqnarray*}f(\vec{x})=\text{sign} \left( \sum _{i\in S}{y}_{i}{\alpha }_{i}^{\ast }\kappa \left( {\vec{x}}_{i},{\vec{x}}_{j} \right) +{b}^{\ast } \right) ,\end{eqnarray*}

respectively. Some commonly-used kernels are as follows:

• Linear kernel: $\kappa (\vec{u},\vec{v})=\vec{u}\cdot \vec{v}.$
• Polynomial kernel: $\kappa (\vec{u},\vec{v})=(\vec{u}\cdot \vec{v}+r)^{n}$, where *r* and *n* are the polynomial coefficient and degree, respectively.
• Radial Basis Function (RBF) kernel: $\kappa (\vec{u},\vec{v})={e}^{- \frac{\parallel \vec{u}-\vec{v}{\parallel }^{2}}{2{\sigma }^{2}} }$, where *σ* is an arbitrary constant.

Among these three kernel functions, the RBF kernel is the most versatile and preferred, especially when not much of the data is known (Sok & Taing, 2014). The numerical results in Tautu & Leon (2012) showed that RBF fared well compared to other kernels in classifying handwritten characters.

## Collecting Baybayin Dataset

Data preparation is the main part of the system. In this section, we discuss how all the necessary dataset for Baybayin, its accents, and Latin characters were collected and compiled. In implementing essential functions to generate the dataset, MATLAB (vR2018a) Image Processing Toolbox was used.

For uniformity of dataset, each image was converted into binary data using a modified *k*-means clustering. *K*-means clustering provides a simple and flexible technique in grouping image intensities. It gives a good performance even in dark images and operates at a low computational complexity since it reduces the data dimension (Pourmohammad, Soosahabi & Maida, 2013). It chooses *k* centers so as to minimize the total squared distance between each point and its closest center. The number of centroids is equal to the number of clusters.

**Figure 5:**
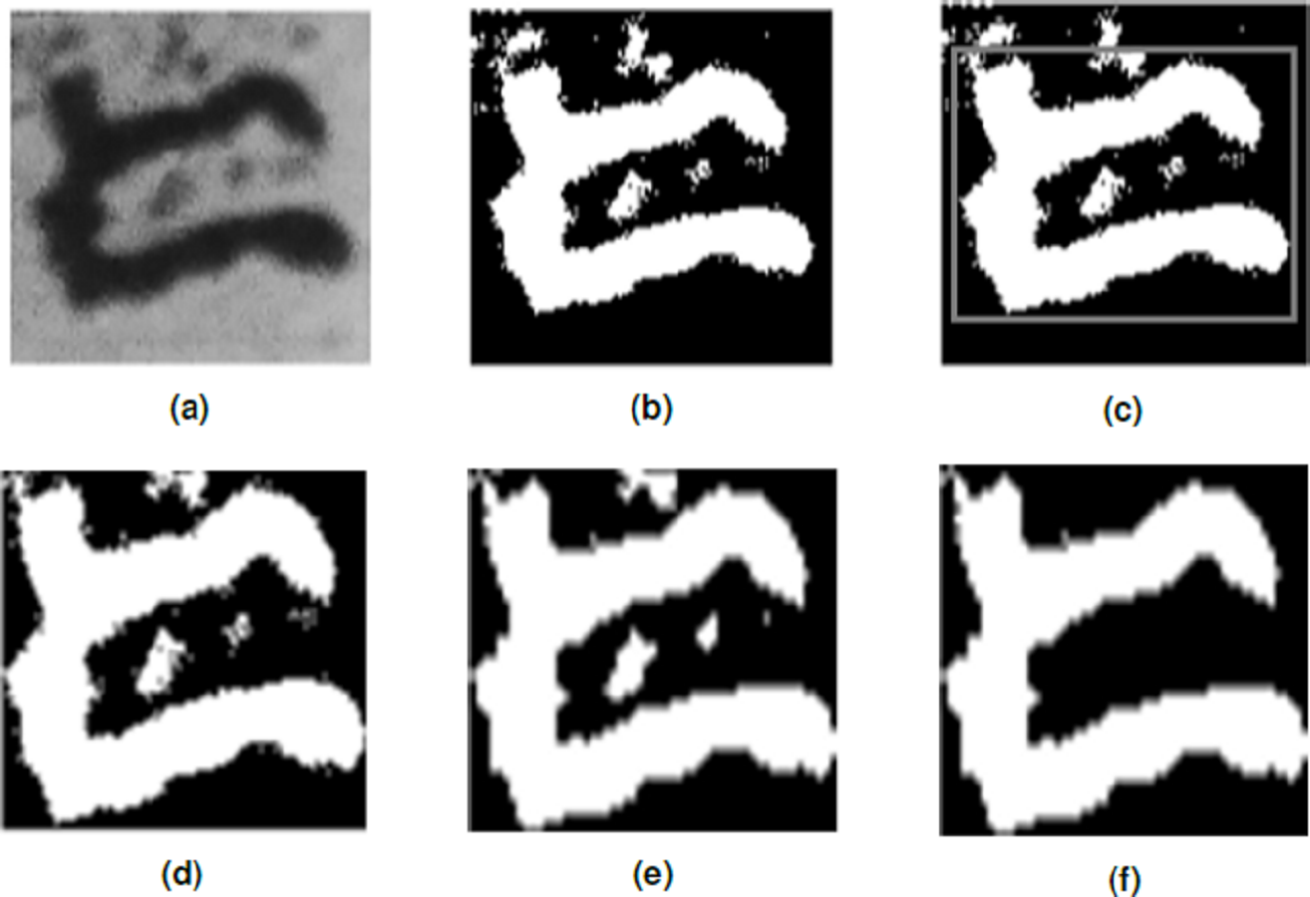
Feature extraction process of a raw character image. (A) Raw character image, 106 × 108; Binary image of A, 106 × 108. (C) Character bounding box, 106 × 108. (D) Cropped image, 86 × 99. (E) Resized image, 56 × 56. (F) Denoised image, 56 × 56.

In this study, *k* = 2 was used in the modified *k*-means function for image binarization. The input image was clustered into two intensities, where lower intensities were set to 1s (white pixels), while higher intensities were set to 0s (black pixels). The MATLAB function regionprops was implemented to provide measurements for each connected component in the input binary image, and the following output were utilized: area, bounding box, and centroid. The image associated with the bounding box was then cropped using the imcrop function to limit the data into its significant features. The cropped image was then rescaled to 56 × 56 pixels using imresize function. To denoise the resized image, the command bwareaopen was used. This command removes all regions that have less pixels than the set pixel value. Finally, the image’s feature vector was generated and then compiled for SVM training. Algorithm 1 and Fig. 5 illustrate the data preparation process succinctly.

For our dataset collection, the images of each Baybayin and Latin characters were searched online. A total number of 9,000+ images for Baybayin characters were taken from the dataset provided by Nogra (2019) in Kaggle. However, some characters in this dataset have less number of images than the other characters. Thus, to complete the dataset, additional images from various websites and books were collected through the use of a snipping tool. Computer-generated and (noisy) handwritten images were also added, as long as the character and its features are visible. The number of images per character was set to 1,000, and after binarization, Algorithm 1 was implemented to each of the binarized images.

Meanwhile, the images for Latin characters were taken from Vial (2017) and Nano (2016) in Kaggle. The generated Latin dataset was set to 700 images per character, with a balanced number of upper and lower cases. The binarized images were also preprocessed using Algorithm 1.

Baybayin diacritic images were also gathered for classification training. Images for dots, bars, cross, and x were collected from Nano (2016) in Kaggle. The images for minus, plus, and times symbols, together with the letter “o”, were modified to produce the diacritic symbols. The binarized images also underwent Algorithm 1 for preprocessing, but due to the characters’ thinness, the imdilate function was applied after the bwareaopen operation to dilate the binary input and provide a preferable data than the original image. A total of 500 images for each diacritic was produced.

The generated dataset for the Baybayin and Latin characters and Baybayin diacritics can be accessed in Pino (2020a).

## Proposed OCR System

The proposed system assumes that the character input has the following properties:

• The character print has the lowest intensity (darkest) than any other part of the image.
• If an input is a Baybayin character with a diacritic or accent, the main body (character) has the largest number of pixels in the image, followed by the accent.
• The accent symbol should be written properly (i.e., the accent symbol should be above or below the main character, should not be touching the main character, and must be within the width of the main character).

**Figure 6:**
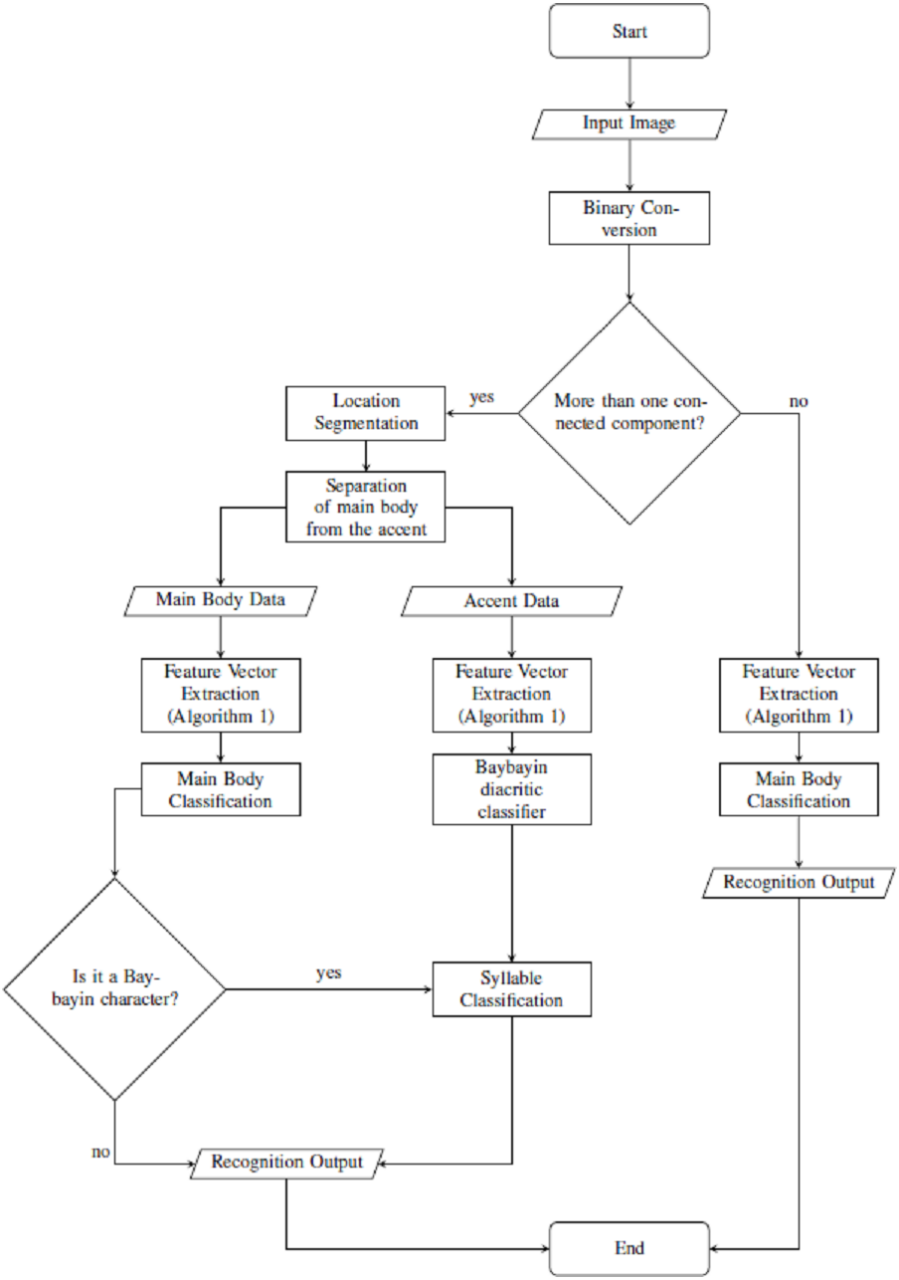
Proposed OCR system.

From an input character image, the system starts with a preprocessing part where the original image is transformed into binary data. If the input has only one component (see Fig. 2A), the system proceeds as in Algorithm 1. On the other hand, if the input has an accent (Figs. 2B to 2D), we locate and separate the main component from the accent/diacritic component, then both components are preprocessed using Algorithm 1. With this, the rest of the structure of the system is constructed from location segmentation, component normalization, feature extraction, classification, and character recognition. Figure 6 shows the overall flow of the system.

### Location segmentation and feature extraction

This is trivial for the one-component image. For the two-component image, after the binarization of the original image using modified *k*-means, the function regionprops was implemented to obtain the information on the image components. Then, the imcrop function was applied to extract the two highest area components (main body and accent). The two components were then isolated. Steps 3 –5 of Algorithm 1 were then implemented to the (isolated) components to obtain their respective 1 × 3,136 feature vectors, which will later be used for classification.

**Figure 7:**
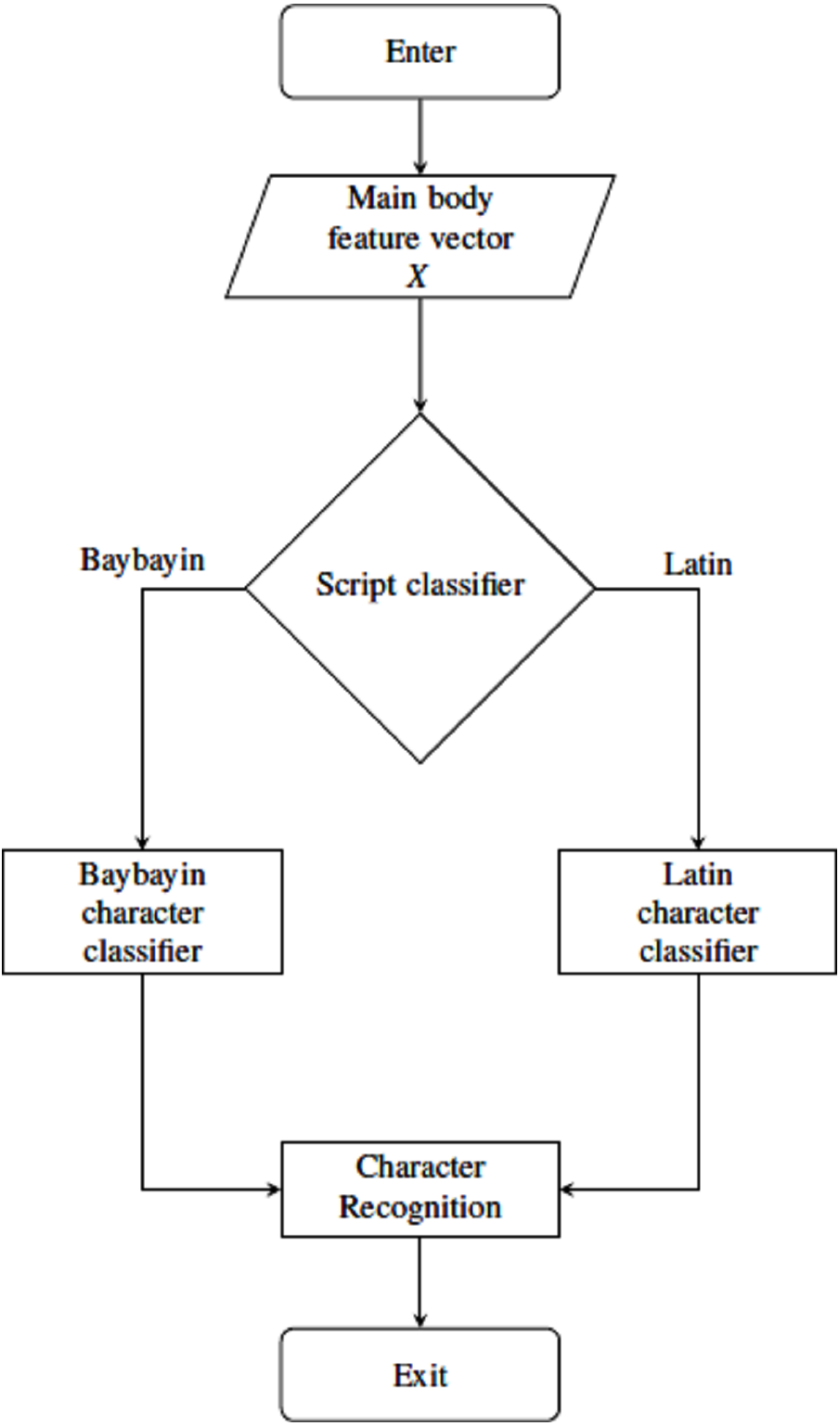
Main body classification process using SVM classifiers.

### Character classification and recognition

For classification and recognition processes, four main SVM classifiers were considered. The main body feature vector *X* goes through two classification nodes as shown in Fig. 7. The first classifier discriminates the Baybayin from the Latin script. Afterwards, if the first classifier determines *X* to be in Baybayin alphabet, *X* proceeds to the Baybayin character classifier and identifies which character unit it represents. A similar method applies if *X* is recognized as a Latin character by the first classifier node. In the case where there is more than one component, the system assumes that *X* is a Baybayin accent (see Fig. 6), and its feature vector goes to the Baybayin diacritic SVM classifier.

#### One component case

From the result obtained in the main body classification, if the image is classified as a Baybayin character, the system prints out its Latin equivalent. On the other hand, if the image is classified as Latin, the Latin character itself is printed out.

#### Two components case

In the two components case, the system separates the main body of the character image from its diacritic (or accent), and identifies the main body as either Baybayin or Latin character.

**Figure 8:**
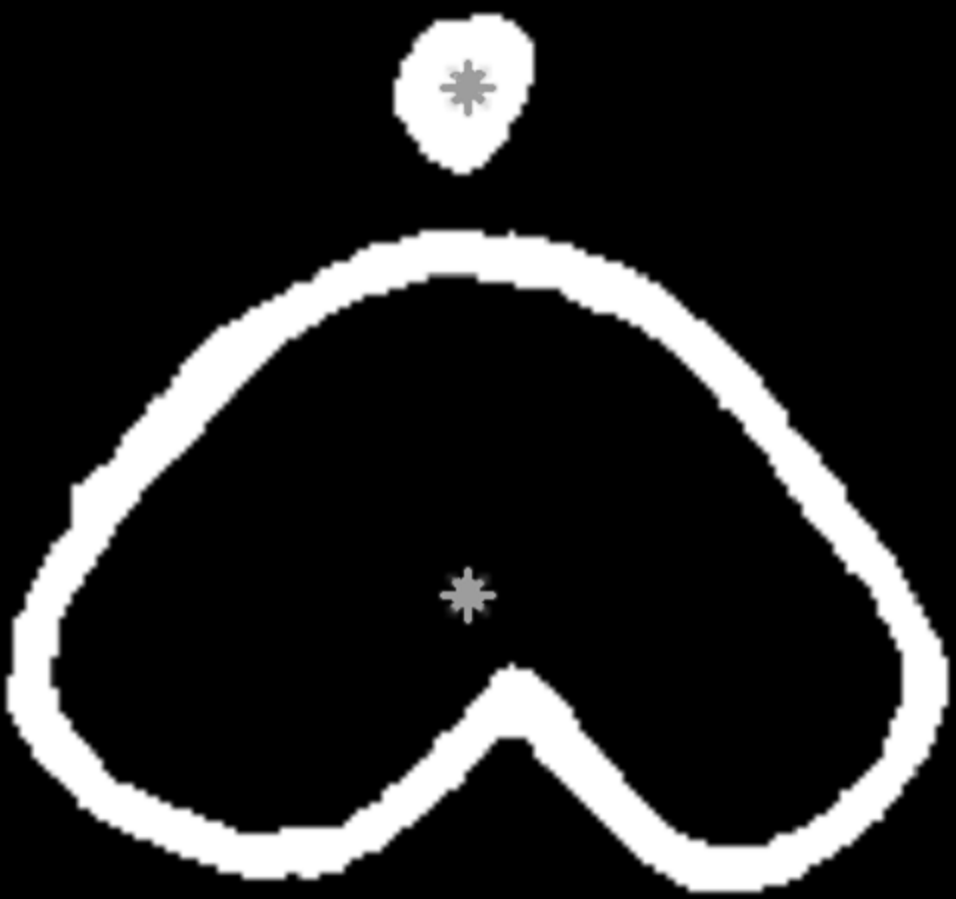
Binary image with the centroid locations superimposed.

If the main body is classified as a Baybayin character, the diacritic classifier result is combined with the Baybayin character for syllable classification. The system determines the correct placement of the accent through its ordinate values from the centroid’s coordinates. That is, if the accent’s centroid ordinate value is less than the main body’s, then its placement is above the character (see Fig. 8). On the other hand, if the accent’s centroid ordinate value is greater than the main body’s, then its placement is below the character. The Baybayin diacritic classifier then discriminates between the dot-bar and cross-x symbol. Finally, the system prints out the Latin equivalent of the Baybayin character with the recognized associated unit represented by the accent.

Meanwhile, if the main body is classified as a Latin character, then there is a chance that a noise was mistaken as another component or it could be that the character is either an ‘i’ or a ‘j’. In the latter case, the system ignores the accent classifier and prints directly the identified Latin character.

### OCR system main process

This section summarizes the main process of the proposed system. Given an individual Baybayin or Latin character input image, the system converts the input into a binary image. Afterwards, if the system detects only one region/component from the input, it will proceed directly to Algorithm 1 for feature extraction process which serves as input for the main body classification. The main body classification process contains SVM classifiers that will categorize the input vector as either Baybayin or Latin, and further recognize it as to what character it represents. The system then outputs the corresponding default Latin syllable for Baybayin characters and an equivalent Latin letter for Latin characters.

For the two component case, the system assumes that the other region represents the Baybayin diacritic symbol. After converting the raw input image into binary data, the system locates the two components’ centroids and computes for their respective region of interest (bounding box). Each of these components then goes through a similar process as in Algorithm 1. The resulting feature vector for the main body proceeds to the main body classification while the accent’s feature vector will be fed into the Baybayin diacritic SVM classifier. If the main body classification results to a Latin script, the system ignores the Baybayin diacritic classifier result and outputs directly its equivalent Latin letter. Otherwise, the Baybayin character is combined with the diacritic information, the placement of which is based on the centroid and the diacritic classifier result. The system then outputs the equivalent Latin syllable or unit it represents. Algorithm 2 summarizes the overall OCR process. The MATLAB code used in this work can be accessed in GitHub shared by Pino (2020b).

## Experimental Setup, Results and Discussions

The SVM models presented here were obtained using the MATLAB (vR2018a) Statistics and Machine Learning Toolbox. The two main functions used were fitcsvm for binary classification and fitcecoc for multiclass classification. Both tools support predictor data mapping with the use of kernel functions, and can employ SVM solvers like Sequential Minimal Optimization (SMO) which is a fast algorithm for training SVMs (Platt, 1998). Moreover, fitcecoc also provides multiclass learning by producing an error-correcting output codes (ECOC) classifier that combines binary classifiers in order to solve a multiclass problem (Escalera, Pujol & Radeva, 2010).

Each SVM model was produced using the template properties shown in Table 1. From the dataset collected, the function fitcsvm was implemented to train an SVM classifier for discriminating Latin and Babayin characters. Training an SVM classifier to differentiate dot and bar from cross and x for the Baybayin diacritics classifier was similarly carried out. An SVM multiclass classification model was trained for each script (Baybayin and Latin characters) using the fitcecoc function. The experiments were carried out with 20% and 30% holdout option for testing, i.e., 80% and 70% of the gathered data were used to train the models.

**Table 1:** Templates used in MATLAB SVM training.

**Property Name/Set to**	**Setup Description**
Kernel Function/‘rbf’	The software makes use of Gaussian kernels in implementing the algorithm to generate a classification model.
Data Standardization/‘true’	The software standardize the predictors before training the classifier for a potential decrease in classification error.
Box Constraint Parameter/‘inf’	The software makes a strict classification which means there will be no points misclassified in training.
Kernel Scale Parameter/‘auto’	The software applies an appropriate kernel norm to compute the Gram matrix that arises from kernel functions.

**Figure 9:**
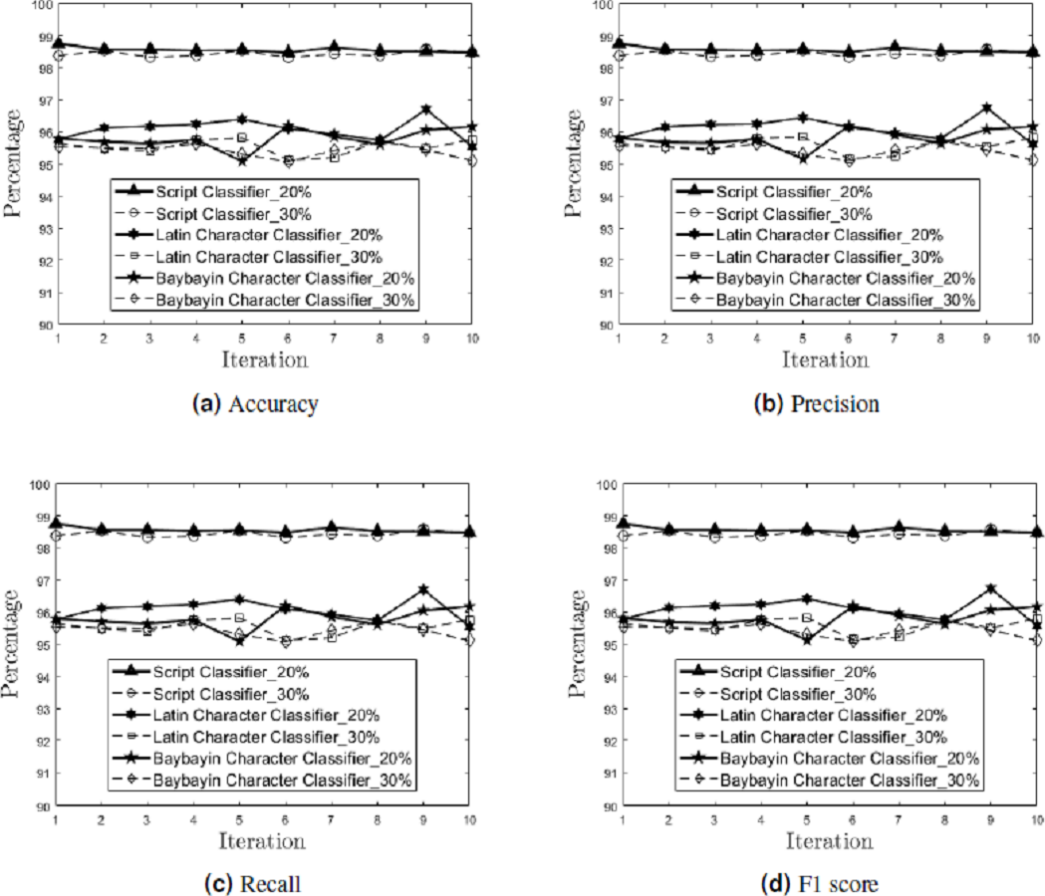
Generalization Performance Results. The generalization performance (accuracy (A), precision (B), recall (C), and F1 score (D)) of each classifier (script, Latin character, and Baybayin character) is measured per iteration, for 20% and 30% holdout samples.

In the multiclass setting, the model’s performance on each character was measured. Figure 9 shows the performance results (accuracy, precision, recall, and F1 score) on each of the ten independent runs for each classifier (script, Latin character, and Baybayin character) in the testing phase, for 20% and 30% holdout samples.

Meanwhile, the generalization performance for the Baybayin diacritics showed 0% classification error in both holdout options. This result can be attributed to the symbols’ unique structures compared to other characters.

The mean and standard deviation of the generalization performance results were also calculated for each holdout options (see Table 2). Empirical results show that the 20% holdout percentage yields better generalization performance than the 30% holdout percentage.

**Table 2:** Mean and standard deviation (SD) of the generalization performance results for 10 iterations in the testing phase with 20% and 30% holdout options. Better performance results between the two holdout options are styled in bold.

Classifiers	Holdout	Accuracy		Precision		Recall		F1 Score	
	Percentage	Mean	SD	Mean	SD	Mean	SD	Mean	SD
Script	20%	**98.56**	0.08	**98.56**	0.08	**98.55**	0.08	**98.56**	0.08
	30%	98.44	0.09	98.44	0.09	98.43	0.09	98.43	0.09
Baybayin Characters	20%	**95.79**	0.32	**95.81**	0.31	**95.79**	0.32	**95.80**	0.32
	30%	95.43	0.21	95.43	0.20	95.43	0.21	95.43	0.21
Latin Characters	20%	**96.07**	0.34	**96.11**	0.34	**96.07**	0.34	**96.10**	0.34
	30%	95.54	0.24	95.58	0.24	95.54	0.24	95.56	0.24

It is noteworthy that most of the errors came from the characters with similar structures. For example, small letter ’m’ and capital letters ‘I’ and ‘T’ from Latin were sometimes recognized as Baybayin characters ‘Na’, ‘Ka’, and ‘La’, respectively (see Fig. 1). Also, in Latin character identification, most errors came from letter ’I’ being identified as either ‘L’ or ‘J’.

**Figure 10:**
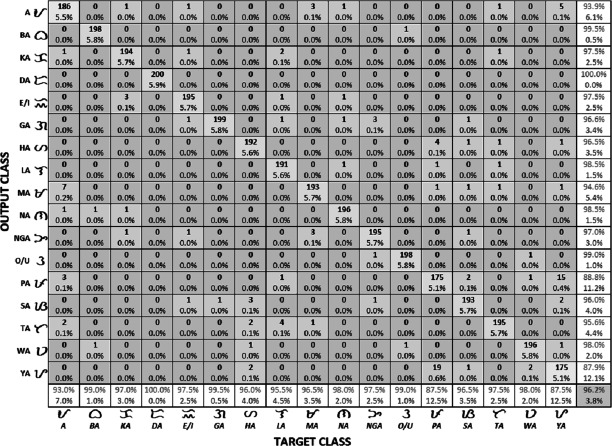
Generated confusion matrix from the best Baybayin character classifier. The diagonal entries correspond to the number of correctly classified characters, while the off-diagonal entries correspond to the number of incorrectly classfied characters. The last column and the last row correspond to the precision and recall of each character, respectively. The bottom right entry is the overall accuracy of the model.

**Figure 11:**
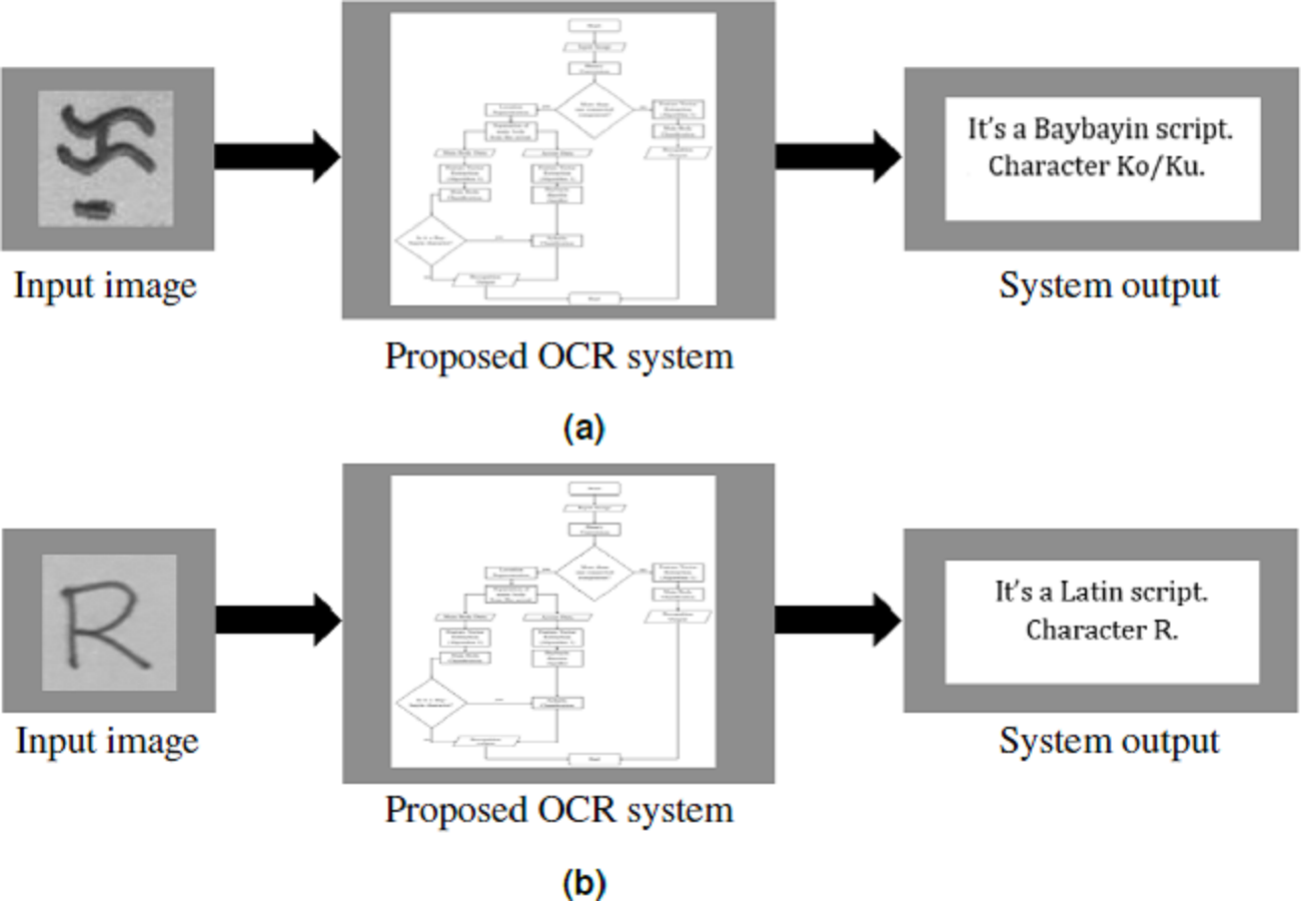
System framework for Baybayin and Latin characters. (A) Character Ko/Ku enters the system. (B) Letter R enters the system.

Figure 10 reports the model’s accuracy (diagonal cells), precision (rightmost column), and recall (bottom row) for each Baybayin character. It can be observed that misclassifications occured among the characters ‘Pa’, ‘Ma’, ‘Ya’, and ‘A’, since they have very similar forms (see Fig. 1). To solve this problem, a reconsideration classifier was added to the main body classification process for these ambiguous characters. The best models were obtained with their respective accuracy: ‘A’ vs. ‘Ma’ –97.75%; ‘Ka’ vs. ‘E’/‘I’ –99%; ’Ha’ vs. ‘Sa’ –99.5%; ‘La’ vs. ‘Ta’ –99%; and ‘Pa’ vs. ‘Ya’ –94.50%. Figure 11 shows an example of Baybayin and Latin character images that are fed into the proposed system and produced a correct classification result (see Figs. 1 and 2 for verification).

To further test the performance of the proposed system on Baybayin characters, the images that satisfy the system’s assumptions (see ‘Proposed OCR System’) were selected from the gathered dataset in Nogra (2019). Among the selected images, 1,100 were randomly chosen for the test runs and were fed into the proposed OCR system for recognition. The conducted experiment obtained an overall average of 98.41%, 98.68%, 98.45%, and 98.57% for accuracy, recall, precision, and F1 score, respectively.

## Conclusions and Future Works

This study has largely contributed to the script and character recognition community by providing a new set of data for Baybayin characters, its diacritics, and Latin characters that can be used in future related studies. This paper also introduced an OCR system that utilizes SVM in discriminating Baybayin script from Latin, and in classifying each of the writing system’s characters. A limitation of this study is that the Baybayin characters written in a manner where the diacritics are attached to the main body were not considered. This setting is sensible since Baybayin characters are generally written with the diacritics detached from the main body. An advantage of this limitation though is that the number of classes in the SVM multiclass algorithm was significantly reduced.

Another strong point of this study is that the proposed system accommodates the highly similar structures among the Baybayin characters, yielding higher generalization performance. Experimental results also show that SVM can be an effective tool in Baybayin character recognition.

For future works, one can explore the use of multiclass SVM in classifying Baybayin characters where the characters with accents are treated as separate classes. One can also explore other machine learning algorithms to solve the classification problems arising from the proposed OCR. A comprehensive comparative study of different classification algorithms applied to Baybayin scripts is also an interesting research direction. Similar to what was shown in Phangtriastu, Harefa & Tanoto (2017), other feature extraction algorithms can be combined with SVM to identify which will work well in classifying Baybayin scripts.

Improving the number of images in the dataset may also significantly decrease the classification error. The Baybayin characters considered in this study are based on the traditional scripts only. Modern Baybayin characters are being proposed recently to conform with the modern Filipino alphabet. Inclusion of these modern Baybayin characters in the proposed OCR system is another interesting extension of this study.

## Additional Information and Declarations

### Data Availability

The following information was supplied regarding data availability: The data used in this article can be found at Kaggle. Specifically: - Baybayin images: https://www.kaggle.com/rodneypino/baybayin-and-latin-binary-images-in-mat-format?select=Baybayin
- Latin images: https://www.kaggle.com/rodneypino/baybayin-and-latin-binary-images-in-mat-format?select=Latin
- Baybayin diacritics images: https://www.kaggle.com/rodneypino/baybayin-and-latin-binary-images-in-mat-format?select=Baybayin+Diacritics
The MATLAB code is available in GitHub:
https://github.com/rbp0803/An-OCR-System-for-Baybayin-Scripts-using-SVM.
